# Earlier de-isolation of SARS-CoV-2-infected ICU patients using a novel viability PCR: a prospective cohort study

**DOI:** 10.1128/spectrum.01140-25

**Published:** 2025-12-05

**Authors:** Una Vojinović, Tom Schoenmakers, Ruben Deneer, Mathie P.G. Leers, Frank van Rosmalen, Stefan H. M. Gorissen, Wilhelmine P. H. G. Verboeket-van de Venne, Walther N. K. A. van Mook, Bas C. T. van Bussel, Paul Savelkoul, Inge H. M. van Loo, Petra F. G. Wolffs, On behalf of the CoLaIC-consortium

**Affiliations:** 1 Department of Medical Microbiology, Infectious Diseases and Infection Prevention, Maastricht University Medical Center + 199236 , Maastricht, the Netherlands; 2 Department of Clinical Chemistry & Hematology, Zuyderland Medical Center, Sittard-Geleen/Heerlen, the Netherlands; 3 School of Nutrition and Translational Research in Metabolism (NUTRIM), Maastricht University 5211, Maastricht, the Netherlands; 4 Department of Intensive Care Medicine, Maastricht University Medical Center + 686882 , Maastricht, the Netherlands; 5 Department of Clinical Chemistry, Catharina Hospital 3168, Eindhoven, the Netherlands; 6 Faculty of Biomedical Engineering, Eindhoven University of Technology 3169, Eindhoven, the Netherlands; 7 Department of Environmental Sciences, Faculty of Science, Open Universiteit, Heerlen, the Netherlands; 8 Cardiovascular Research Institute Maastricht (CARIM), Maastricht University 5211, Maastricht, the Netherlands; 9 Zuyderland Academy, Zuyderland Medical Center, Sittard-Geleen/Heerlen, the Netherlands; 10 School of Health Professions Education (SHE), Maastricht University 5211, Maastricht, the Netherlands; 11 Academy for Postgraduate Medical Training, Maastricht University Medical Center + 686882 , Maastricht, the Netherlands; 12 Care and Public Health Research Institute (CAPHRI), Maastricht University 5211, Maastricht, the Netherlands; Universidade Federal do Rio de Janeiro, Rio de Janeiro, Brazil

**Keywords:** de-isolation, viability PCR, SARS-CoV-2, COVID-19

## Abstract

Intensive care unit (ICU) patients with COVID-19 are isolated until two subsequent SARS-CoV-2 PCR tests have become negative. The question is whether the viral RNA detected here represents intact or compromised viral particles. A viability PCR method detects only intact infectious viruses and disregards RNA from incomplete non-infectious viruses or RNA remnants. We hypothesize that the time to a negative PCR is shorter using the viability PCR compared to conventional SARS-CoV-2 PCR. Patients admitted to the ICU in the Maastricht University Medical Center within an ongoing prospective cohort were included, and respiratory samples were collected three times weekly. Samples were aliquoted for viability PCR into two aliquots, one of which was treated with propidium monoazide (PMA) to eliminate incomplete viruses. The PMA-untreated sample corresponded to the traditional SARS-CoV-2 PCR used in routine diagnostics. A mean time difference between viability and conventional PCR was estimated, assuming a negative binomial distribution. One hundred two ICU patients were included in the analysis. The time to a negative PCR test result differed significantly between viability PCR and conventional PCR (Paired Prentice-Wilcoxon test *P*-value = 0.0001). On average, the first negative viability PCR test result occurred 2.4 days (95% CI 1.5–4.2) before the first negative conventional PCR test result. The application of a viability PCR technique for SARS-CoV-2 provided an earlier indication of the absence of intact virus, suggesting reduced infectivity, potentially enabling earlier de-isolation of ICU patients.

**IMPORTANCE:**

This study demonstrated that, within patients, on average, viability PCR became negative 2.4 days earlier than conventional PCR, indicating that viability PCR could potentially assist in de-isolating patients 2.4 days earlier. Future use of this assay could thus aid in improving routine COVID-19 diagnostics and prognostics related to the timing of de-isolation. Additionally, future development of a similar approach for other respiratory viruses could be of interest.

## INTRODUCTION

The prevention of SARS-CoV-2 transmission by adequate isolation measures remains a laborious task for clinicians and hospital infection preventionists. More importantly, isolation measures can have unfavorable consequences on the quality of care for patients and their overall well-being (1). Real-time polymerase chain reaction (RT-PCR) assays are the gold standard method for laboratory diagnosis of SARS-CoV-2 and other viruses. However, RT-PCR testing cannot discern between non-infectious and infectious viruses. This is especially problematic when patients have persistently positive RT-PCR results, and the dilemma arises as to whether this represents a true replication-competent virus or remnant viral RNA. Such persistently positive PCRs have been observed in patients for weeks and sometimes even months (2–4). Several other studies indicated that this could be due to the detection of remnant viral RNA (5–7). Unnecessarily prolonged isolation measures can cause a burden on patients, intensive care unit (ICU) personnel, ICU isolation room capacity, and healthcare costs.

The need for a diagnostic tool capable of detecting intact viruses instead of remnant RNA is evident. Although viral culture has traditionally been used for this aim, this method is not widely applied as a diagnostic tool in routine practice in many clinical microbiology laboratories. One technique that utilizes membrane integrity as the main aspect of viability is termed viability PCR (v-PCR) (8). This method utilizes pre-treatment of the sample with a membrane-impermeant nucleic acid stain, followed by RNA extraction and amplification. Propidium monoazide (PMA), which solely penetrates compromised membranes, irreversibly intercalates in the viral RNA and thereby inhibits the amplification of PMA-altered RNA (9). Since the RNA from viable virus is left unmodified, only RNA from intact virus will be amplified in subsequent analyses. Compared to PMA, PMAxx is a novel and improved viability dye that can be utilized for the distinction between infectious and non-infectious pathogens (10).

A v-PCR assay for the detection of SARS-CoV-2 in patient samples has recently been developed and thoroughly validated by (11, 11). This study found that this v-PCR assay can discern intact, infectious SARS-CoV-2 from damaged, non-infectious SARS-CoV-2 in cultured virus samples as well as in a clinical sample. Within this validation study, the v-PCR showed that PMAxx treatment effectively blocks more than 99.99% of RNA from compromised SARS-CoV-2 virions, only amplifying RNA from intact SARS-CoV-2 virions (12). Sberna et al. (12) used a v-PCR in conjunction with a digital droplet PCR, while the v-PCR by (11, 11) uses RT-PCR. The latter is more often used in determining the SARS-CoV-2 virus. The current study focuses on assessing the potential implications for earlier de-isolation in the ICU. Therefore, the v-PCR based on RT-PCR was preferred based on the current clinical practice.

We hypothesize that the time to negative PCR test conversion is shorter using the v-PCR method, and isolation duration can potentially be shortened using v-PCR compared to conventional PCR.

## MATERIALS AND METHODS

### Design and Study Population

The Maastricht Intensive Care COVID (MaastrICCht) cohort has been described extensively elsewhere (13–15). In brief, this comprehensive prospective cohort study was conducted in patients admitted to the ICU of the Maastricht University Medical Center+, a tertiary care university teaching hospital in the southern part of the Netherlands (13). The MaastrICCht cohort has been ongoing and reported on mechanically ventilated patients until 1 October 2021 (16). For the current study, we investigated an additional 109 patients with and without mechanical ventilation admitted to the ICU from 1 October 2021 to 1 May 2023. During this period, nasopharyngeal swab sampling was performed every Monday, Wednesday, and Friday for RT-PCR and v-PCR analyses. These repeated samples are considered repeated measurements within the same patient.

PCR sampling stopped when PCR results were no longer considered clinically indicated by a joint decision of the attending ICU clinicians and microbiologist. A distinction was made between two types of patients: those whose ICU admission was directly related to COVID-19 (“primary SARS-CoV-2 infection”) and those whose SARS-CoV-2 infection was coincident with warranted ICU admission (“non-primary SARS-CoV-2 infection”).

### Outcome measures

The time to negativity was determined for both the PMA-treated as well as the PMA-untreated aliquots of each patient sample. The time to negativity was defined as the number of days from ICU admission until the first PCR with a Ct-value of 35 or higher. In this study, the day of ICU admission was the starting day (T0).

### Sample collection and preparation

Respiratory samples were taken with nasopharyngeal swabs in a viral transport medium. ICU sample collection was done three times weekly for each patient. On the day of sampling, the nasopharyngeal swabs were transported to the laboratory for further processing.

Each sample was split into three aliquots: one for treatment with PMAxx (labeled + PMA), one left untreated (labeled—PMA), and one for the PCR assay used in routine diagnostics. A total of 250 µL of the sample was pipetted in two 1.5 mL tubes, while 1 mL of the sample was pipetted in the tube meant for the PCR assay used in routine diagnostics.

To the aliquots for PMAxx treatment, 2.5 µL 0.005% SDS solution (Sigma-Aldrich) and 1 µL 20 nM PMAxx (Biotium Inc., Hayward, CA) were added. Hong et al. showed that adding surfactants such as SDS improved differentiating power and v-PCR, in particular, the addition of 0.005% SDS (17). These tubes were vortexed and incubated for 30 min at 37°C. For PMAxx to be activated and bind RNA of inactivated SARS-CoV-2 virus particles, the PMAxx-containing tubes were placed in the PMA-lite LED photolysis device for 10 min. A total of 250 µL of Lysis buffer was added subsequently. A total of 250 µL of Lysis buffer was added directly to the tubes containing samples that were left untreated.

### Virus RNA extraction and RT-qPCR conditions

The RT-qPCR conditions were the same for both PMAxx-treated and PMAxx-untreated samples for the v-PCR method. The only difference was that for the PMAxx-treated PCR procedure, samples were pre-treated with PMAxx, SDS, and photoactivation. Samples for the PMAxx-untreated PCR method were not.

SARS-CoV-2 RNA was isolated using the MagNA Pure LC 96 DNA and viral RNA NA Small Volume Kit (Roche) using the Pathogen Universal 200 protocol.

The E-gene was targeted using the forward primer 5′-CGGAAGAGACAGGTACGTTAATAG-3′, reverse primer 5′-AGACCAGAAGATCAGGAACTCTA-3′, and probe 5’−6-FAM-ACACTAGCCATCCTTACTGCGCTTCG-BHQ-1-3’.

RT-qPCR was run on a QuantStudio 5 device (Applied Biosystems, Thermo Fisher Scientific, Waltham, MA) with primers and probes targeting the SARS-CoV-2 E-gene. Ct values were assessed with Quantstudio Design and Analysis Software v1.5.2. Each RT reaction was performed by adding 10 µL of viral RNA to a mix containing 5 µL Taqpath 1-step RT qPCR MasterMIX (Applied Biosystems, Thermo Fisher Scientific, Waltham, MA) and 5 µL primer-probe mix. Amplification was done by a 2 min hold stage at 25° and a hold stage for 15 min at 50°. This was followed by 42 cycles of 3 s at 95°C and 30 s at 60°C.

To correctly evaluate whether v-PCR becomes negative earlier compared to conventional PCR, Ct 35 and above was considered as a negative test result. Using this cut-off value, we aimed to measure time-to-negativity more accurately by avoiding the variability of Ct value outcomes caused by the on-off effect of samples with a low viral load.

### Statistical methods

Patient characteristics were summarized using mean and standard deviation for normally distributed variables and median and interquartile range (IQR) otherwise. The outcome was the time (in days since ICU admission) until a negative PCR (viability and/or conventional) test result. The data were considered paired since the conventional and v-PCR tests were performed on the same patient/sample (i.e., no biological variability, with two tests, each with potential analytical variability), while multiple serial samples per patient (i.e., taken on Monday, Wednesday, and Friday) were investigated. In addition, the outcome is considered right-censored because sampling stopped when patients were discharged from the ICU or died (which could occur before a negative test result, indicating censoring).

To assess whether there was a significant difference in the time-to-negativity between the v-PCR and conventional PCR test, the Paired Prentice-Wilcoxon (PPW) test for censored paired data was used (18). Furthermore, the 50% event time (the time at which half of the population was negative for the corresponding PCR) was also assessed. To plot the different times to negativity between v-PCR and conventional PCR, a cumulative incidence plot was used. A two-sided test with a *P*-value < 0.05 was considered significant.

To calculate the average number of days that a v-PCR test result was negative before a conventional PCR test result, we first calculated the time difference between a negative viability test result and a negative conventional PCR test result for each patient. If the conventional PCR test result was negative before the v-PCR test result, the time difference was <0; if the viability test result was negative before the conventional test result, the time difference was >0. Since the time to negative data is censored, the differences are also censored. If a patient was discharged after a negative v-PCR test result, but the last conventional PCR test result was still positive, the difference was right censored. To account for censoring in the differences, parametric distributions were fitted to the data using maximum likelihood estimation. A normal, Poisson, and negative binomial distribution were fitted, and the best-fitting distribution was chosen based on the Akaike Information Criteria and Bayesian Information Criteria (BIC) of the fitted distributions. For the best-fitting distribution, the mean and the corresponding 95% CI are reported. The confidence interval is estimated via bias-corrected and accelerated bootstrap method with 2,000 resamples.

To further investigate the results, a post-hoc analysis was performed. In this sub-analysis, the effects of primary/non-primary SARS-CoV-2 infection, chronic lung disease, immunosuppression, and variant of concern on the difference between v-PCR and conventional PCR were further investigated (Supplement 1).

All analyses were performed in R version 4.1.2 (19), and the fitdistrplus package version 1.2-1 (20) was used to fit the different distributions to the censored differences in time to a negative PCR test result.

## RESULTS

The study population consisted of 109 patients, of whom 69 (63.3%) were admitted with a primary SARS-CoV-2 infection and 40 (36.7%) with a non-primary SARS-CoV-2 infection. The median age of patients was 64 years (IQR 56, 72), with a median body mass index of 26 (IQR 24.7, 30.9) kg/m^2^. The median length of stay before the ICU admission was 1 (IQR 0, 5) day; the length of stay in the ICU was 11 days patients; 74 (67.9%) survived their ICU stay (Table 1).

**TABLE 1:** Descriptive statistics of the cohort^*a*^

Variables	Units		Overall
Number of patients		*N*	109
Sex	Male	*N* (%)	78 (71.6)
	Female	*N* (%)	31 (28.4)
Age	Years	Median (IQR)	64 (56, 72)
BMI	Weight (kg)/height (m^2^)	Median (IQR)	26.7 (24.7, 30.9)
ICU length of stay	Days	Median (IQR)	11 (5, 21)
Pre-ICU length of stay	Days	Median (IQR)	1 (0, 5)
ICU mortality	Survivor	*N* (%)	74 (67.9)
	Non-survivor	*N* (%)	35 (32.1)
Primary or non-primary COVID	Primary COVID	*N* (%)	69 (63.3)
	Non-primary COVID	*N* (%)	40 (36.7)
APACHE II score	Clinical score	*N* (%)	14 (11, 18)
Chronic lung disease	Yes	*N* (%)	22 (20.2)
	No	*N* (%)	87 (79.8)
Immunosuppression	Yes	*N* (%)	7 (6.4)
	No	*N* (%)	102 (93.6)

^*a*^ APACHE, Acute Physiology and Chronic Health Evaluation; BM, body mass index.

**Fig 1:**
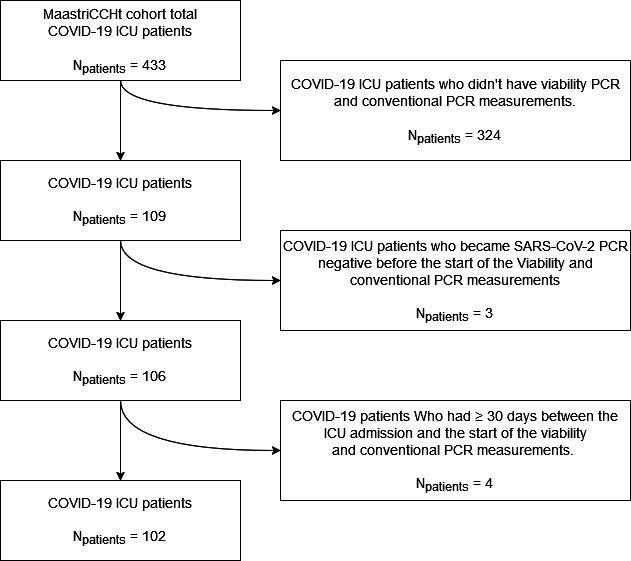
Cohort inclusion scheme inclusion flowchart of the cohort. v-PCR sampling started at the start of October 2021, resulting in a subset of 109 patients within the MaastrICCht cohort. The subset of 102 patients was analyzed in this study.

Of the 109 patients, seven were excluded because of a negative PCR result between T0 and the start of the first v-PCR measurements or no PCR measurement for 30 or more days between the T0 and first v-PCR measurements, leaving 102 patients for the analysis (Fig. 1).

Within the 102-patient cohort, the number of days to PCR negativity ranged from 0 to 31 days for the conventional PCR and from 0 to 20 days for the v-PCR. The median number of days to a negative PCR result was 5 (IQR 2, 12) days for conventional PCR and 4.5 (IQR 2, 9) for the v-PCR.

Of the 102 patients, 70 (68.6%) had a negative result for the v-PCR during ICU stay, and 32 (31.4%) stayed positive for the v-PCR during the ICU stay and thus were considered censored. For the conventional PCR, 62 patients (60.8%) had a negative result during the ICU stay, and 40 patients (39.2%) stayed positive.

The 50% event time for negativity (time point where half of the PCR became negative had occurred) was 7 days post-admission (95% CI: 5–10) for v-PCR and 11 days (95% CI: 8–15) for conventional PCR.

**Fig 2:**
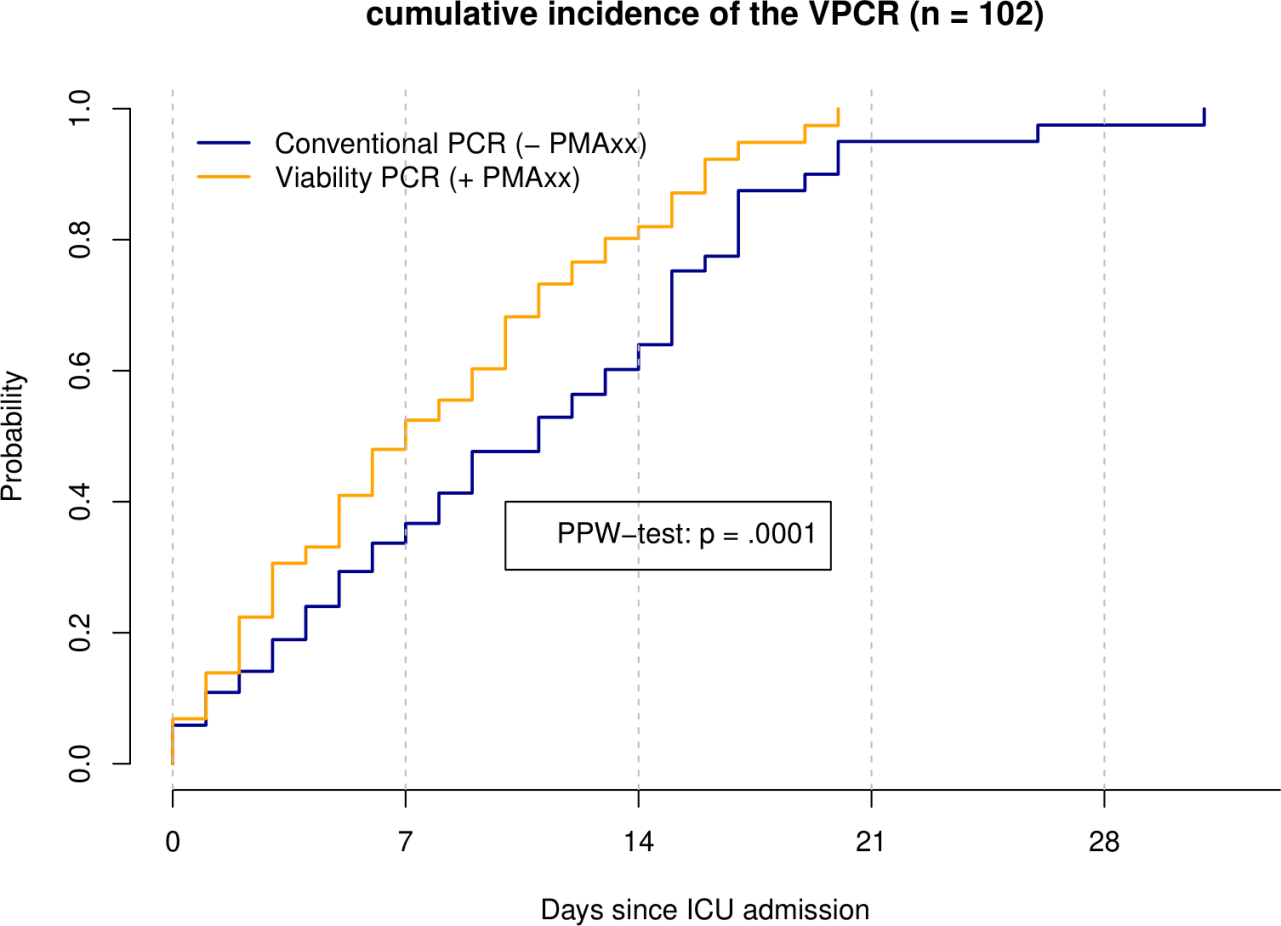
Cumulative incidence of the conventional and v-PCR becoming negative in time. This figure shows the cumulative incidence plot of both the v-PCR (orange line) and conventional PCR (blue line) becoming negative in time. The plot shows that the v-PCR becomes negative earlier in comparison with the conventional PCR. This difference between the tests was statistically significant as assessed via the PPW test (*P* = 0.0001).

The difference between the time-to-negativity of the v-PCR and conventional PCR was statistically significant (*P* = 0.0001) as assessed by the PPW-test (Fig. 2). This is a strong indication that the occurrence in time of PCR negativity is different for v-PCR and conventional PCR. In only one case did the conventional PCR become negative before the v-PCR (v-PCR Ct: 34.2, conventional PCR Ct: 35.4). In the other cases, the v-PCR became negative before the conventional PCR became negative. There were no significant differences measured for primary/non-primary SARS-CoV-2 infection, chronic lung disease status, immunosuppression status, and variant of concern on the difference between v-PCR and conventional PCRs (Supplement 1).

**Fig 3:**
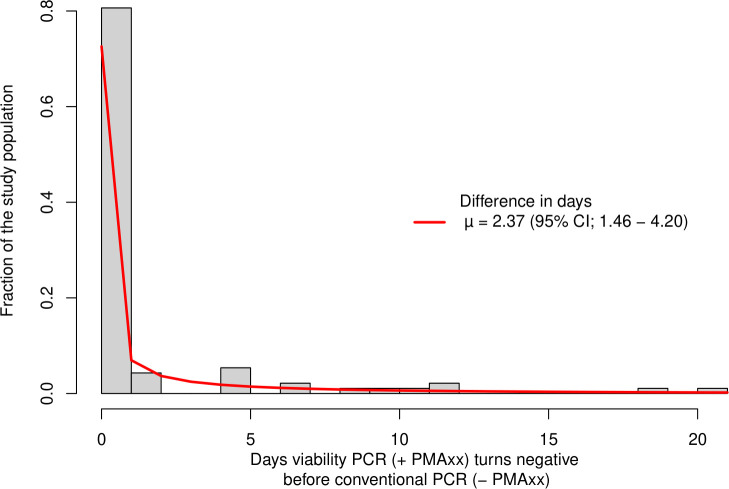
Distribution of the time difference between the v-PCR (+PMAxx) and conventional (−PMAxx) PCR becoming negative. This figure shows the time difference distribution between the test becoming negative. Population density is shown on the y-axis, and on the x-axis, the difference in days between the conventional PCR and v-PCR negativity is shown. If they both became negative on the same day, this would be zero, and if the v-PCR was negative first, a positive time difference is shown. A negative-binomial distribution (red line) was fitted on the data to assess the mean (µ) time difference (2.4 days [95%; CI 1.5–4.2] earlier).

To estimate the magnitude of the difference, the distribution of the differences had to be modeled using the best-fitting distribution. The negative non-binomial distribution had the best fit according to the BIC score and visual residual inspection. Using this fitted distribution, the average difference was calculated. The resulting average difference in days was 2.4 days (95% CI; 1.5–4.2), in which the v-PCR converted earlier to negative compared to the conventional PCR assay (Fig. 3).

## DISCUSSION

The main finding of this study is that in ICU patients with SARS-CoV-2, the time-to-negativity of the v-PCR is, on average, 2.4 days shorter compared to the currently routinely used conventional PCR assay. Our findings thus confirm the hypothesis that pre-treatment of samples with PMAxx shortens the detection time of PCR negativity since only the intact virus is consequently detected.

This evidence contributes to the optimization of the decision-making approach regarding discontinuation of isolation measures for patients admitted with COVID-19. Regular PCR assays cannot discern between intact and remnant viruses, and thus between infectious and non-infectious viruses. This can lead to dilemmas for clinicians regarding patients with persistence, for example, beyond 10 days, positive PCR test results. Our findings imply that with v-PCR testing, isolation measures could be discontinued earlier. Theoretically, the 2.4 days earlier patients’ de-isolation (as implied by the results of this study) could substantially reduce the burden on patients, ICU personnel, ICU isolation room capacity, as well as healthcare costs.

Although the benefit of 2.4 days to de-isolate earlier is clinically relevant, based on earlier reports, the expected difference in time-to-negativity is expected to be greater. This can be explained by several aspects:

1. Suboptimal pre-treatment efficiency with PMAxx could be an underlying cause for longer detection of viral RNA remnants. Veugen et al. demonstrated a blocking efficiency of over 99% in both viral culture samples and clinical samples (11). Since we have applied the same assay protocol from the latter study, suboptimal efficiency seems a less plausible explanation, though we have not analyzed assay efficiency in our present study.
2. Second, the method employed in our viability assay is based on the assumption that a compromised cell membrane is the underlying mechanism leading to reduced virus viability. As previously mentioned by Veugen et al. (11), it could be argued that a compromised cell membrane could only be one of multiple possible mechanisms of reduced virus viability and, therefore, not the only measure of viral viability. Furthermore, there is also a possibility of the free-floating RNA (indicative of non-intact viral particles) being encapsulated in extracellular vesicles. This would also lead to a slightly diminished effect of the v-PCR.
3. Third, PCR samples were collected three times per week, not daily. This could imply that some samples became negative within this interval, underestimating the effect of the v-PCR.
4. Last, it could be a sign of longer persistence of the replication-competent virus in patients than hypothesized (21). A previous study investigated prolonged persistently positive RT-PCR cases using a subgenomic viral mRNA detection method for assessment of viral viability. This study found that among cases with prolonged viral shedding, subgenomic viral RNA was detected in 20% of cases long after symptom onset (22). This report has underscored that prolonged conventional PCR positivity should not be automatically dismissed as detection of remnant RNA, as it might indicate true infectiousness in some patients. In other terms, a prolonged PCR could indicate true prolonged infectiousness.

Regarding our post-hoc exploration, several remarks should be made. In the initial analyses, we did not include information regarding the viral variant types. Additional sequencing of the samples that were collected in our study could give more insights into this in-host evolution phenomenon. We thus considered whether viral integrity is dependent on viral variants. Though our post hoc exploration should be interpreted cautiously, the distribution of the data shows no clear indication supporting this assumption (Supplement 1). Similarly, a previous report did not find relevant differences in the time to a negative culture outcome for Delta and Omicron variants (23). Concerning an immune-compromised status, one study reported no prolonged virus shedding in a cohort of patients, illustrating that this does not necessarily have to be a factor, implying a longer presence of viable virus (24). Furthermore, additional analyses show that the initial viral load does not seem to significantly affect (*P* = 0.37) the time difference variability between the viability and regular PCR (Supplement 2).

Our analysis has certain limitations. First, we used the day of ICU admission as the first time point since information regarding SARS-CoV-2 PCR tests taken elsewhere from patients who were admitted to the ICU was lacking. This could have impacted the time to PCR negativity and most likely underestimated the potential gain in days by v-PCR. Second, the duration of PCR positivity for the v-PCR assay could be overestimated since samples were not taken daily but three times per week. Despite these limitations, a clinically relevant 2.4-day reduction was observed.

Third, it has to be noted that in this study, we have not tested samples in parallel using another—gold-standard—detection method capable of detecting viable viruses (viral culture). Therefore, we cannot state with absolute certainty that the shedding of infectious viruses is equal to zero. Nevertheless, the v-PCR used in this study has been validated using culture as a gold standard, as published elsewhere (11). Last, we used Ct 35 and above as a cut-off for negativity to assess time-to-negativity more accurately. In current clinical practice, there is no consensus on the use of a Ct threshold value for making decisions regarding infectivity and isolation duration.

Apart from these limitations, this study also has several strengths. First, repeated measurements were done within patients of both conventional and v-PCR until negativity. Combined with simultaneous testing of v-PCR and conventional PCR, this gave an accurate measure of the difference between the two PCR tests in time. Second, the need to isolate, and consequently the need for testing infectivity, is present in patients who are admitted with primary and non-primary SARS-CoV-2 infection, while the latter group has not been admitted to the ICU for COVID-19 and thus may not have this disease. By including both patients with primary and non-primary SARS-CoV-2 infection, the generalizability of the results was enhanced. Last, the currently chosen study design and utilization of PCR are highly representative of clinical practice reality during the SARS-CoV-2 pandemic.

This study demonstrated that within patients, on average, v-PCR became negative 2.4 days earlier when compared with conventional PCR, indicating that using v-PCR could potentially assist in de-isolating patients 2.4 days earlier. Future use of this assay could thus aid in improving routine COVID-19 diagnostics and prognostics related to the timing of de-isolation. Additionally, future development of a similar approach for other respiratory viruses could be of interest.

## Data Availability

The raw data can be obtained upon reasonable request from the corresponding author.

## SUPPLEMENTAL MATERIAL

Checklist spectrum.01140-25-s0001.docx STROBE checklist for cohorts.

Supplemental material spectrum.01140-25-s0002.pdf Supplements S1 and S2.

Research data spectrum.01140-25-s0003.xlsx Ct values and data dictionary.

ASM does not own the copyrights to Supplemental Material that may be linked to, or accessed through, an article. The authors have granted ASM a non-exclusive, world-wide license to publish the Supplemental Material files. Please contact the corresponding author directly for reuse.
