# Supplementary material for: Earlier de-isolation of SARS-CoV-2-infected ICU patients using a novel viability PCR: a prospective cohort study
Source: Microbiol Spectr. 2025 Dec 5;14(1):e01140-25. doi: 10.1128/spectrum.01140-25 (PMC12772352; doi:10.1128/spectrum.01140-25)
Supplement: Supplemental material — Supplements S1 and S2. [file spectrum.01140-25-s0002.pdf]

Supplement 1: A posthoc analysis

Based on the findings in our primary analysis and outcome data, a further post-hoc exploration was done. We aimed to ascertain what patients could benefit mostly from viability-PCR testing. Covariates were defined using the ICU admission characteristics and whether a pathophysiological link could be expected. These processes could be linked to the patient not clearing the viral debris, which could explain the differences observed between the viability PCR and conventional PCR.

Two outcome groups were made: patients with no difference between viability PCR and normal PCR were defined as the 0 group, and patients with a difference as the 1 group. Logistical regression analysis was then performed with the variants, primary/ non-primary SARS-CoV-infection status, immunosuppression status, and chronic lung disease status as covariates related to the outcome variable.

Within the total 102 patients, 69 had primary COVID-19, seven patients were immunocompromised (non-immunocompromised: 95 patients), 19 patients had chronic lung disease (no chronic lung disease: 83 patients), and finally, 40 patients had the Delta variant and eight the Omicron variant, 54 patients could not be genotyped and were classified as unknown for the variant of concern. The confidence intervals around the odds ratios of the covariates indicated that no significantly different odds ratios were found for the group of patients that had a difference or hadn't had a difference in days between the viability and normal PCR (table S1).

Thus, within this study, no univariate covariate could be found as a clear explanation for the difference between the groups, which did or did not show a difference between the time to negative for viability and conventional PCR. The odds ratio estimation for primary COVID-19 status, chronic lung disease, and immune suppression was consistent with expectations.

However, this post-hoc analysis should be interpreted with caution. The lack of statistical significance suggests that the observed odds ratio may be due to random variation. Furthermore, the original goal of this study was not designed to ascertain the covariates that explain the observed difference or lack thereof. These additional results should, therefore be interpreted with caution.

Supplement Table 1: Logistical regression dichotomous difference time to negativity between viability and normal PCR (Y= 1 difference between PCRs, Y=0 no difference between PCRs)

	$Y_{binary\ difference\ PCRs}$			$Y_{binary\ difference\ PCRs}$			$Y_{binary\ difference\ PCRs}$			$Y_{binary\ difference\ PCRs}$		
Predictor	Odds Ratio	95% CI	p	Odds Ratio	95% CI	p	Odds Ratio	95% CI	p	Odds Ratio	95% CI	p
(Intercept)	0.18	0.06 – 0.42	< .001	0.22	0.12 – 0.37	< .001	0.23	0.14 – 0.38	< .001	0.38	0.18 – 0.74	.006
Non-primary/primary SARS-CoV-2 infection (0/1)	1.56	0.54 – 5.18	.435									
Chronic lung disease				1.62	0.47 – 5.00	.417						
Immunosuppression							1.71	0.23 – 8.67	.54			
Variant [Omicron]										2.64	0.54 – 13.04	.22
Variant [unknown]										0.38	0.11 – 1.16	.101
Observations	102			102			102			88		
R2 Tjur	.006			.007			.004			.071		

Variants unknown: Samples that could not be genotyped due to high CT values were categorized as unknown.

Supplement 2: Effect of the initial viral load on the time difference variability between the viability and regular PCR

To assess the variability in time-to-negativity among patients with comparable viral load we first grouped the patients based on the first regular PCR test within this study (this would give a rough estimation of the viral load) in comparable viral load groups as follows: patients with a Ct above 30 were grouped (group: Ct > 30), and patients with a Ct lower than or equal to 30 were another group (group: Ct ≤ 30).

Those two groups were used to investigate the variability in time to negativity. Therefore, the same steps were taken to calculate the average difference in days as elaborated in the manuscript. This resulted in two histograms, one for the group with Ct >30 (Supplemental figure 1) and the other for the group with Ct ≤30 (Supplemental figure 2).

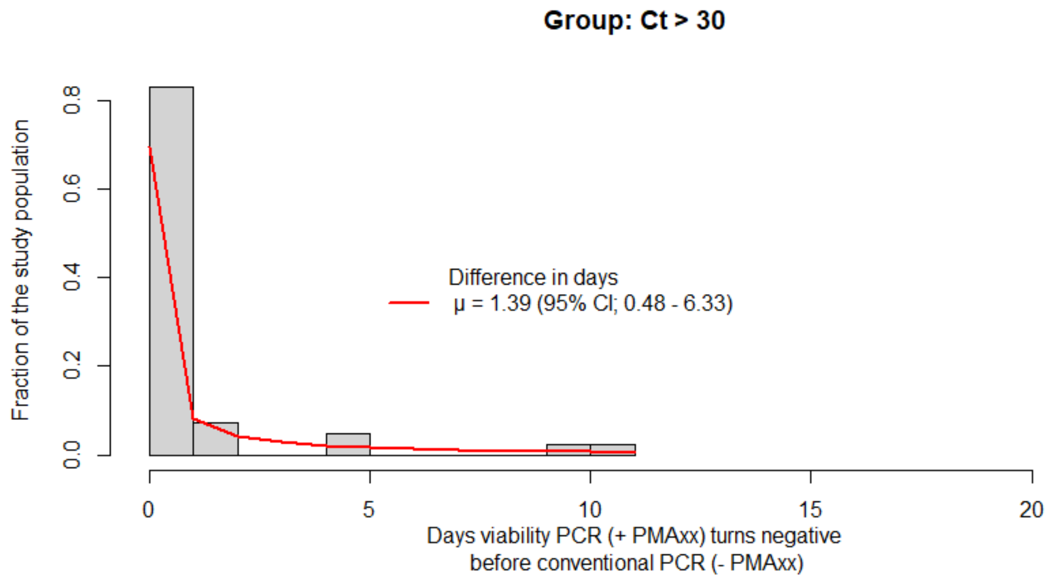

Supplemental figure 1: Distribution of the time difference between the viability (+PMAxx) and conventional (-PMAxx) PCR becoming negative for the group: Ct > 30 (Initial.

This figure shows the distribution of time differences between the test becoming negative. Population density (fraction of the study population) is shown on the y-axis, and on the x-axis, the difference in days between the conventional PCR and viability PCR negativity is shown. If they both became negative on the same day, this would be zero, and if the viability PCR was negative first, a positive time difference is shown. A negative-binomial distribution (red line) was fitted on the data to assess the mean (μ) time difference (1.39 days (95%; CI 0.48 – 6.33) earlier)

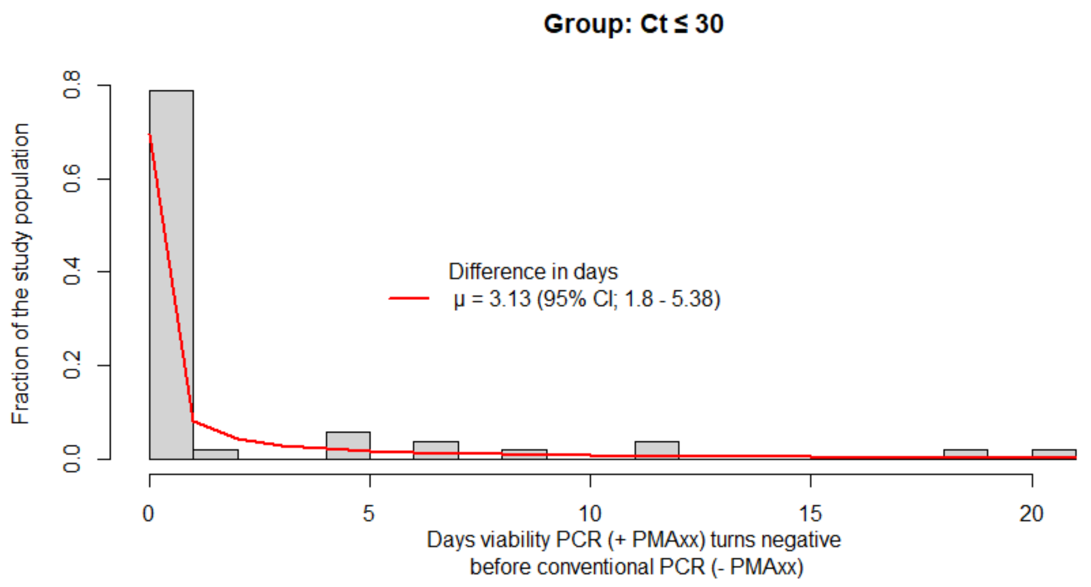

Supplemental figure 2: Distribution of the time difference between the viability (+PMAxx) and conventional (-PMAxx) PCR becoming negative for the group: Ct ≤ 30.

This figure shows the distribution of time differences between the test becoming negative. Population density is shown on the y-axis, and on the x-axis, the difference in days between the conventional PCR and viability PCR negativity is shown. If they both became negative on the same day, this would be zero, and if the viability PCR was negative first, a positive time difference is shown. A negative-binomial distribution (red line) was fitted on the data to assess the mean (μ) time difference (3.13 days (95%; CI 1.80 – 5.38) earlier).

Time-to-negativity differs between the two groups, with a point estimate of 1.39 and 95% Confidence interval (CI) of 0.48 - 6.33 days in the first group and 3.13 and 95% CI of 1.80 - 5.38 days in the other group. A Levene's test was performed on this data to investigate the variability between the two. This test showed that the variability was not statistically different between the two groups (P = 0.37).

This is further corroborated by the confidence interval in which there is an overlap between the group: Ct > 30 (95% CI: 0.48 – 6.33) and the group: Ct ≤ 30 (95% CI: 1.8 – 5.38). furthermore, the confidence intervals of the mean time difference are also broader when compared to the full cohort analysis. This is likely due to the lower number of patients in the initial Ct group categories compared to the full cohort.
